# Impact of Design and Deployment Technique on the Hydrodynamic Resistance of Flow Diverters: An in Vitro Experimental Study

**DOI:** 10.1007/s00062-021-01106-1

**Published:** 2021-10-22

**Authors:** Dániel Gyürki, Benjamin Csippa, György Paál, István Szikora

**Affiliations:** 1 Department of Hydrodynamic Systems, Faculty of Mechanical Engineering, Budapest University of Technology and Economics, Műegyetem rkp. 3., D building, 3rd floor, 1111 Budapest, Hungary; 2 Department of Neurointerventions, National Institute of Clinical Neurosciences, Budapest, Hungary

**Keywords:** Flow diverter, Stent, Hydrodynamic resistance, Experimental set-up, Stent deployment

## Abstract

**Purpose:**

Despite the high efficacy of flow diverters (FD) in treating sidewall intracranial aneurysms, failures are reported. One of the physical factors determining efficacy is the flow reducing capacity of the FD that is currently unknown to the operator. Our aim was to measure the flow reducing capacity expressed as the hydrodynamic resistance (HR), the metallic surface area (MSA) and pore density (PD) of two different FD designs and quantitatively investigate the impact of sizing and the deployment technique on these parameters.

**Methods:**

Altogether 38 Pipeline (Medtronic) and P64 (Phenox) FD‑s were implanted in holder tubes by a neurointerventionist in nominally sized, oversized and longitudinally compressed or elongated manners. The tubes were placed in a flow model with the flow directed across the FD through a side hole on the tube. HR was expressed by the measured pressure drop as the function of the flow rate. Deployed length, MSA and PD were also measured and correlated with the HR.

**Results:**

Both PD and MSA changed with varying deployment length, which correlates well with the change in HR. Oversizing the device by 1 mm in diameter has reduced the HR on average to one fifth of the original value for both manufacturers.

**Conclusion:**

This study demonstrates experimentally that different FD designs have different flow diverting capacities (HR). Parameters are greatly influenced by radial sizing and longitudinal compression or elongation during implantation. Our results might be useful in procedure planning, predicting clinical outcome, and in patient-specific numerical flow simulations.

**Supplementary Information:**

The online version of this article (10.1007/s00062-021-01106-1) contains supplementary material, which is available to authorized users.

## Introduction

Endovascular methods are commonly used to treat intracranial aneurysms [1]. Among those, flow diverter (FD) stent implantation is an effective technique for the treatment of large and broad-necked sidewall aneurysms [2–4]. Despite the higher efficacy of flow diversion compared to coil embolization, some aneurysms still fail to occlude following FD treatment [5, 6].

Beside the patient’s proactive biochemical environment, the efficacy of flow diversion is determined by multiple factors, such as the geometric parameters of the device, and the deployment technique used by the operator. Both of these factors have an impact on the effective flow reducing capacity of the FD, also called as the hydrodynamic resistance (HR) of the implanted device. Of note, in this paper the expression “hydrodynamic resistance” is used for the pressure drop through the mesh of the FD as a function of the flow rate.

One of the key factors determining HR is the porosity of the device. By decreasing the porosity, the resistance increases [7, 8]. The nominal porosity of each device is given by the producer for an unconstrained condition; however, using braided stents, this factor can be modified by the operator to some extent applying various deployment techniques and by selecting the size of the device in relation to the vessel diameter. Deploying the FD with an increased forward push will increase the mesh density and decrease its porosity and final length. Less forward pushing on the other hand results in elongation and higher porosity of the implanted FD [9, 10]. Regarding sizing, it has been shown that FDs with a nominal diameter that matches the diameter of the target vessel produces higher metal coverage and subsequently lower porosity compared to oversized ones. Hence oversizing is expected to decrease the HR between the sac and the parent vessel [11–13].

In vitro measurements and computational fluid dynamics (CFD) investigating the flow patterns in the aneurysm sac have been used to quantify the efficacy of FDs [14, 15]. Complex and valid simulation of both the mechanical behavior of the FD during deployment and its flow effect is desirable to provide guidance for the operator in choosing the most appropriate technique and device [16, 17]. This can be achieved by directly modelling the individual struts of the FD; however, the enormous computational need makes its use in practice questionable [18]. Alternatively, a FD can be simulated as a homogeneous porous layer covering the target vessel from the inside but such simulation necessitates accurate knowledge of the HR of the porous layer [19, 20].

### Purpose

The purpose of our study was to determine the flow reducing capacity of FD stents from various manufacturers expressed as the HR, using different deployment techniques and sizing strategies, and correlate it with geometric parameters of the devices, such as the metallic surface area (MSA) or the pore density (PD). These measurements aim at providing data for porous media-based CFD simulations assisting neurointerventionalists in choosing the appropriate device and technique based on quantitative results.

## Methods

Every solid object placed in a flow presents a hydrodynamic resistance that is manifested in a pressure loss, which originates from two physical mechanisms. One is the friction resistance stemming from the direct viscous friction on the walls of the body. Second, the so-called form resistance, stemming from the uneven pressure distribution on the front and back side of the body, caused by flow separation. At low flow velocities typically the friction resistance dominates (linear term in Eq. 1) and at high velocities the form resistance (quadratic term in Eq. 1).

**Fig. 1:**
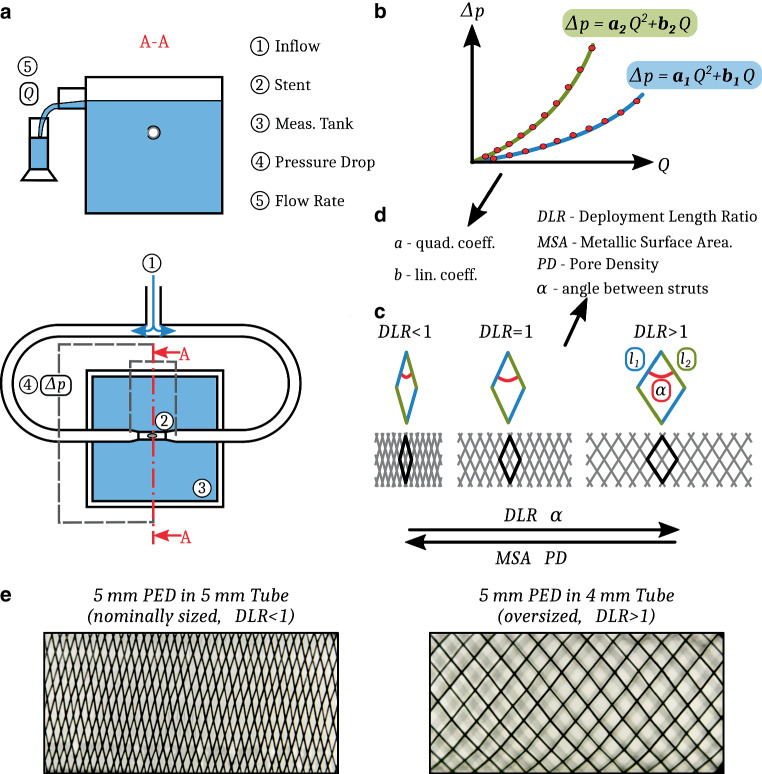
**a** Sketch of the measurement rig, **b** two different HR curves, **c** effect of compression or elongation on the braided stents, *l*_1_and *l*_2_ are the side lengths, *α* is the angle of the rhombus, **d** list of acquired values from the measurements, **e** microscopic images of a nominally sized and an oversized PED device mesh.

For the measurements, we developed an experimental rig to model the flow through the FD stents. Details of the measurement rig and that of the procedure can be found in Csippa et al. [21]. Fig. 1a shows the sketch of the measurement rig. The FDs were placed in a tailor-made transparent holder tube with an elliptic hole in the side. The tubes were placed within a flow loop, the flow passing through the side hole across the FD. The pressure drop and the flow rate through the FDs were measured. The resistance curve is a quadratic function in the following form

1 $$\Delta p=aQ^{2}+bQ,$$

where *Δp*[*Pa*] is the pressure drop on the mesh, $Q\left[ml/s\right]$ is the volumetric flow rate, $a\left[Pas^{2}/ml^{2}\right]$ is the quadratic coefficient and $b\left[Pas/ml\right]$ is the linear coefficient. The form of this curve is schematically depicted in Fig. 1b for two different set of coefficients, representing different resistances.

For each device, sizing and deployment technique, three sets of data were measured and recorded according to Fig. 1, such as:

- hydrodynamic data including pressure drop on the mesh and flow rate indicating HR (Fig. 1b, d),
- geometrical data of the deployed device including the MSA, the ratio of the wire-covered area to the whole area, the PD$\left[\textit{pores}/mm^{2}\right]$ and the angles between the struts ($\alpha \left[^{\circ}\right]$) using the following formulae (Fig. 1c, d).

2 $$MSA=\frac{\textit{Strut pixels}}{\textit{All pixels}}$$

3 $$\textit{Surface area}=l_{1}l_{2}sin\left(\alpha \right)$$

4 $$PD=\frac{\textit{Number of pores}}{\textit{Surface area}}$$

5 $$DLR=\frac{L_{2}}{L_{1}}$$

- the length of the deployed device (*L*_2_), and the ratio of the deployed- and nominal length (*L*_1_) as the deployment length ratio (DLR), indicating the deployment technique, such as compressed or elongated.

For the pressure drop equation, the quadratic (*a*) and the linear (*b*) coefficients were separately recorded as they may impact HR differently.

The HR curves were generated by fitting a parabolic function to measurement points, as depicted in Fig. 1b. Each FD deployment was measured four times to estimate the measurement uncertainties, and every curve contained around 10–12 points. The devices remained in the holder tubes during a measurement series, therefore the deployment length did not change for the deployment scenario. The linear (*b*) and quadratic coefficients (*a*) were determined by fitting the curve on all the points from the four measurement series (around 40 points).

The geometrical data of the deployed devices were obtained using image processing techniques described in detail in Csippa et al. [21]. The deployed stents at the elliptical hole of the holder tube were photographed using a USB microscope (Dino-Lite, Torrance, CA, USA). A middle rectangular area of the stent was selected in order to avoid the distortion caused by the cylindrical shape. Then, based on Eqs. (2–4), the geometrical data can be calculated using pixel count. This part of the evaluation was done in the open-source software ImageJ version 1.2.4 RRID:SCR_003070 (U.S. National Institutes of Health, Bethesda, MD, USA).

In most cases the mesh of the stent was homogeneous at the hole of the holder tube after implantation. Even if not, the evaluation methods of the geometrical data and the coefficients of the HR average the results for the whole hole on the holder tube.

Two different FD designs were used from two manufacturers (Pipeline Embolization Device, Medtronic, Minneapolis, MN, USA: referred to as PED; P64 flow modulation device, Phenox, Bochum, Germany: referred as P64). FDs of 5, 4 and 3 mm diameters by both manufacturers were measured, referred later as FD5, FD4 and FD3 respectively. The FD stents were deployed in the holder tubes by a neurointerventionist (I. Sz.). We used three holder tubes with diameters of 5, 4 and 3 mm; these tubes are referred later as Tube5, Tube4 and Tube3, respectively.

To estimate the impact of deployment technique, different FDs were deployed into the same sized holder tube using different techniques in terms of longitudinal device compression or elongation resulting in three different deployed lengths. This reflected the variety of techniques used by the operators in clinical practice and the inherent uncertainty of the deployment technique. To test the effect of radial sizing, first we performed measurements with the nominal diameter of the stents matching the diameter of the holder tube (e.g. FD5 in Tube5); these scenarios are called nominal sizing. The effects of oversizing were tested by placing the FD in a 1 mm smaller diameter tube than its nominal diameter (e.g. FD5 in Tube4).

## Results

### Nominal Sizing

All of the measured data can be found in Online Resource 1. Supplementary Table 1 contains the measured data for nominally sized cases, when the diameter of the deployed device matches the diameter of the holder tube. Each number represents the average of 4 measurements consisting of at least 10 different measurement points.

The coefficients of the HR curve are the following. In the case of FD5, *a* and *b* vary between 0.028 and 0.213 Pas^2^/ml^2^, and 1.5 and 4.4 Pas/ml, respectively, taking both manufacturers into consideration. Supplementary Table 1 shows for the FD4 measurements that *a* and *b* change from 0.041 to 0.126 Pas^2^/ml^2^ and from 1.3 to 5.0 Pas/ml, respectively. In case of FD3, the range of *a* is 0.017–0.293 Pas^2^/ml^2^, and of *b* is 3.5–9.3 Pas/ml.

The following geometrical data were obtained from the measurements. The MSA changes from 0.344 to 0.481 in case of FD5. The range of MSA for FD4 is 0.283–0.449, while the MSA for FD3 varies from 0.305 to 0.357. As for the PD, the values range between 18 and 34 pores/mm^2^ in case of FD5. For FD4 the PD varies between 16 and 34 pores/mm^2^. Last, the values for FD3 measurements are in the 17–32 pores/mm^2^ range.

As seen in Supplementary Table 1 in the case of nominally sized FDs, the DLR almost never exceeds 1. This means that as a rule the nominally sized devices are compressed compared to their nominal lengths; however, it is important to notice that the range of DLR for the three nominal diameters are different. The DLR values for FD5 change from 0.58 to 0.85, for FD4 they change from 0.64 to 1.12, while in case of FD3 the range of DLR is 0.83–0.98.

In nominally sized cases the angles vary between 22–37°, 30–47° and 40–56° for FD5, FD4 and FD3 respectively. As other geometrical data, the angles are also dependent on the deployment length.

### Oversizing

Supplementary Table 2 contains the measurement data for the cases, when the FDs were deployed in an oversized manner, so the diameter of the holder tube is 1 mm smaller than the diameter of the device. In case of oversized FD5 measurements, stents with nominal diameter of 5 mm were placed in a holder tube with 4 mm diameter. For oversized FD4 measurements, the oversized stent diameter is 4 mm.

As Supplementary Table 2 illustrates for the FD5 in Tube4 measurements, the coefficients of the HR curve vary between 0.014 and 0.140 Pas^2^/ml^2^ and between 0.11 and 1.01 Pas/ml for *a* and *b*, respectively. These values change from 0.025 to 0.308 Pas^2^/ml^2^, and from 0.75 and 4.66 Pas/ml, respectively, in the case of the FD4 in Tube3 measurements.

The MSA varies between 0.199 and 0.270 in case of oversized FD5 FD‑s, and between 0.205 and 0.275 for the FD4 ones. The range of PD values for FD5 in Tube4 is 10–21 pores/mm^2^, while for FD4 it is 13–19 pores/mm^2^. The angles vary between 54° and 83°, and between 63° and 91°, for oversized FD5 and FD4, respectively.

Last, the DLR values change from 1.19 to 2.03 in the case of the FD5 measurements, and the range for FD4 is 1.44–1.90.

**Fig. 2:**
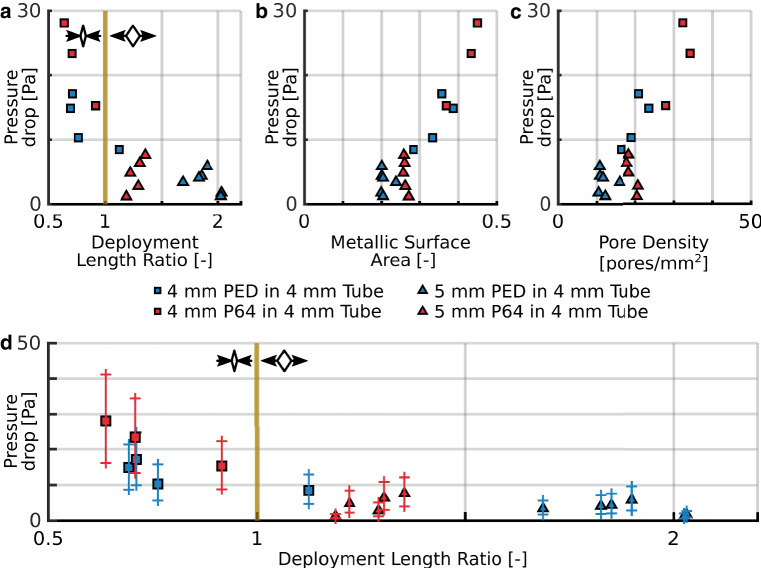
The effects of radial sizing in the case of FD5 and FD4 in Tube4 measurements. The *vertical yellow lines* represent the nominal deployment length, the *small pictograms* represent the longitudinally compressed and elongated deployment scenarios. *Blue* markers correspond to PED, while *red* markers correspond to P64 measurements. *Squares* are the nominally sized and *triangles* are the oversized cases. **a** Pressure drop calculated with 5 ml/s flow rate as the function of the deployment length ratio. **b** Pressure drop calculated with 5 ml/s flow rate as the function of the metallic surface area. **c** Pressure drop calculated with 5 ml/s flow rate as the function of the pore density. **d** Pressure drop calculated with 5, 3 and 7 ml/s flow rate (the mid-point and the two endpoints of the range, respectively, indicated by the symbol and the range around it) as the function of the deployment length ratio

First, we studied the difference between a nominally sized and an oversized FD for a given holder tube, thus the effect of device sizing. Fig. 2 demonstrates these results for Tube4, this means that 5 mm and 4 mm devices (FD5 and FD4) were deployed in Tube4. A similar figure can be found in Online Resource 1 for FD4 and FD3 in Tube3 comparisons. The different graphs show the pressure drop as the function of DLR, MSA and PD. The blue and red markers differentiate between the manufacturers, while the shape of the marker is the distinction between the nominally sized (squares) and oversized (triangles) measurements. In Fig. 2a–c, the pressure drop is calculated with a flow rate of 5 ml/s in order to display the HR quantitatively. In Fig. 2d, the upper limit of the depicted range is calculated with 7 ml/s, while the lower limit is calculated with 3 ml/s, to make the different deployment scenarios comparable in a given range of blood flow rate.

**Fig. 3:**
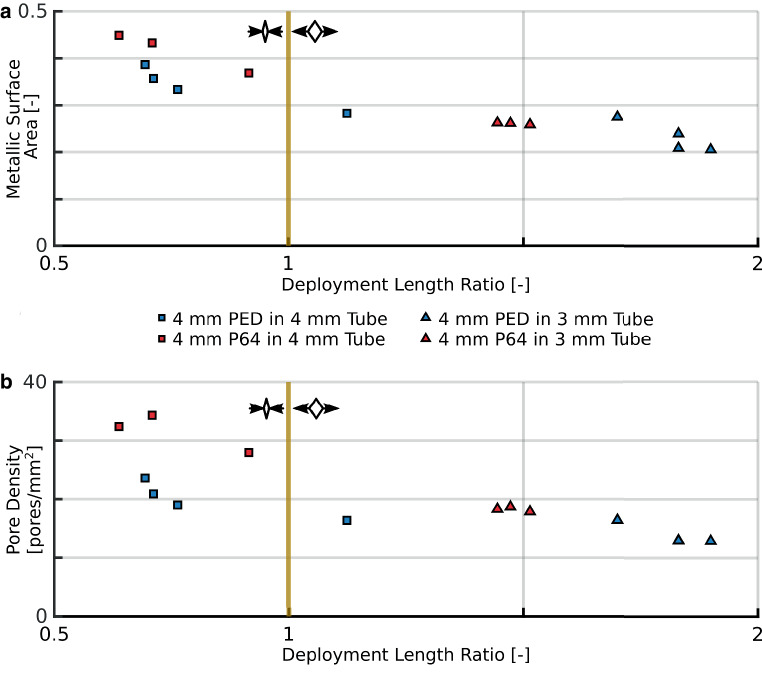
The effects of longitudinal compression or elongation in the cases of FD4 in Tube4 and Tube3 measurements. The *vertical yellow lines* represent the nominal deployment length, the *small pictograms* represent the longitudinally compressed and elongated deployment scenarios. *Blue* markers correspond to PED, while *red* markers correspond to P64 measurements. *Squares* are the nominally sized and *triangles* are the oversized cases. **a** Metallic surface area as the function of the deployment length ratio. **b** Pore density as the function of the deployment length ratio

Next we observed the effect of deployment technique, namely the longitudinal compression or elongation during deployment. We assembled the results of a given device deployed in nominally sized holder tube and in a 1 mm smaller holder tube. Fig. 3 presents the results in case of FD4 deployed in Tube4 and Tube3, but a similar figure can be found in Online Resource 1 for the FD5 in Tube5 and Tube4 comparisons. Fig. 3a, b displays the MSA and PD as the function of the DLR. These graphs visualize the effect of the deployment length on the geometrical parameters.

**Fig. 4:**
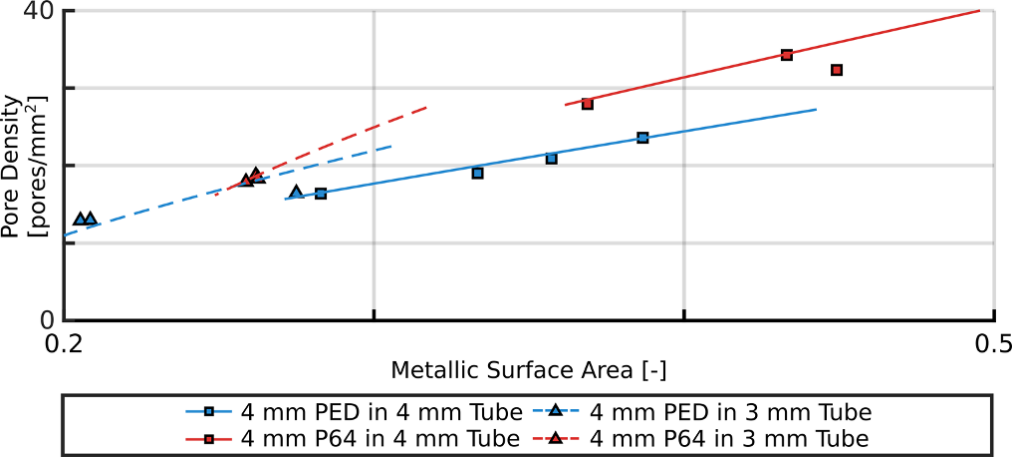
Pore density as the function of the metallic surface area in the cases of FD4 in Tube4 and Tube3 measurements. *Blue* markers correspond to PED, while *red* markers correspond to P64 measurements. *Squares* are the nominally sized and *triangles* are the oversized cases. The *continuous* and *dashed lines* represent the theoretical relationship calculated with devices diameter of 5 and 4 mm respectively

The special braided geometry provides a strong relationship between the geometrical parameters. These relations are further investigated to study the difference between the manufacturers. Fig. 4 presents the measured PD values as the function of the MSA for the FD4 in Tube4 and Tube3 cases. A similar figure can be found in Online Resource 1 for the other cases as well. The lines represent the theoretical relationship between the MSA and PD values, which can be derived due to the braided geometry [22]. Since the theoretical lines depend on the actual device diameter, the continuous lines are calculated with the nominally sized diameter, while the dashed lines are calculated with the 1 mm smaller diameter. This was needed to make them comparable with the respective measurements.

## Discussion

Choosing the optimum device for treating an aneurysm is heavily based on the experience of the medical practitioner. The objective was to quantitatively investigate the relations between the resistance of devices by various manufacturers and in various deployment scenarios. Augsburger et al. found that although the porosity is a key factor in determining the flow reduction capability of the device, it is solely not sufficient [8]. We argue, as in our previous paper that the really decisive factor is the HR [21], of which the porosity is only one element. In this sense our findings complement those of Augsburger et al.

By definition, the FD stent slows down the flow in the aneurysm sac by placing a hydrodynamic resistance between the aneurysm and the parent artery. In our paper, the expression “resistance” is used for the pressure drop through the mesh of the FD. In this research, we obtained the coefficients of Eq. 1, the geometrical parameters of the device, and investigated the effect of radial and longitudinal sizing on these parameters. In contrast with Augsburger et al., who determined these coefficients by auxiliary simulations, in this paper they are determined by measurements.

### Effect of Radial Sizing

Fig. 2 clearly displays the difference between choosing a nominally sized or an oversized device for a given vessel diameter. The FDs with different diameters are separated in Fig. 2a, d with respect to the pressure drop and the DLR. Implanting a device in a smaller holder tube resulted in a much larger deployment length, therefore the DLR of oversized FDs may even reach values higher than two, meaning the deployed length is twice the nominal length of the device.

Based on Supplementary Tables 1 and 2, it can be said that the radial sizing affects the linear coefficient (*b*) of the HR curve, while the quadratic coefficients (*a*) are in the same range for the nominally and oversized stents. As stated before, it is visible in Fig. 2a, d that the pressure drop makes a noticeable jump between the over-sizing and the nominal sizing. These findings further verify the statement that in the physiological flow rate range the linear term dominates the resistance and the magnitude of the quadratic term is of secondary importance. These results suggest that choosing an oversized stent for treating an aneurysm over a nominally sized stent may produce an insufficient resistance through the aneurysm neck, therefore the thrombosis in the sac may not be complete.

This correlates well with the results in Supplementary Table 2, as oversizing clearly increases the *α* angles. In the case of FD5 in Tube4 scenarios, for both PED and P64 devices the values of the nominally sized measurements are 50% on average of the oversized ones, while in case of FD4 in Tube3, this is around 60%. During oversizing a stent, the angle of the rhombi increases, reaching even 90°, so the rhombi become squares, the area of the pores become the largest, therefore the PD and MSA values are the smallest at that point. This correlates well with Wang and Yuan [23] and Aurboonyawat et al. [24]. Our results indicate that the HR reaches its minimum value at that point because there is less solid surface for viscous friction. Oversizing further, the angles would grow over 90°, the squares would become rhombi again, and the PD, MSA and HR values would increase again; however, by oversizing a stent with 2mm (e.g. a 5 mm device in a 3 mm holder tube) the deployed device would cover a longer section of the vessel. It may unnecessarily increase the length of a temporarily thrombogenic metallic surface and the risk of side branch occlusion.

These measurements show that the radial sizing is an important factor of the HR and thus the flow reducing capacity of the deployed device. Based on the results of this research, practitioners could take into consideration the diameter of the chosen device.

### Effect of Longitudinal Sizing

Based on Figs. 2 and 3, it can be said that the longitudinal sizing of a device plays an important role. According to Fig. 3, the deployment length affects the parameters of a stent. Both MSA and PD are dependent on the deployment length, hence the longitudinal compression/elongation. If the DLR is larger, the rhombi of the braiding (pores between the struts) become less and less squeezed. As a result, the pores grow, approaching a square, therefore the area covered by the struts becomes smaller compared to the whole. Similar thoughts can be applied to the relationship between DLR and PD. If the rhombi grow, fewer pores cover the same area, so the PD decreases, as also found by Makoyeva et al. [25], This phenomenon is closely related to the change in HR. If the operator elongates the FD stent, the area of the rhombi increases, the porosity increases as well, so the resistance of the deployed stent is reduced.

Fig. 4 also confirms this unequivocal relationship between the MSA and PD. As expected, by increasing MSA the number of pores on a given area increases also, while the angle between the struts decreases. We note that the finding that the PD increases with the MSA is not a general law but a special feature of the braided geometry. This phenomenon is depicted in Fig. 1c, e. Fig. 4 also displays the theoretical relationship between MSA and PD. The continuous line is the theory calculated with the nominal diameter of the device, which should be compared with the squares, while the dashed line is calculated if the FDs are deployed in an oversized manner and should be compared with the triangles. These graphs show that our results are in good agreement with the theoretical relationship, this confirms the validity of our measurements.

These results suggest that the longitudinal sizing is a major factor in the efficacy of a device, which in the case of braided stents, the operator can control to some extent. For example, across the aneurysm neck, the practitioner could compress the stent to increase the MSA value, and with it the resistance, thus reducing the flow inside the aneurysm sac; however, across the origin of important side branches, a looser deployment might be applied to avoid side branch occlusion [13]. Therefore, choosing the appropriate resistance is of great importance. It should be emphasized that the deployed length of the device is mainly controlled by the diameter of the parent vessel, and in nominally sized devices the deployed length can be varied only in a limited range and further elongating the FD may result in malapposition.

### Effect of Strut Number

The main structural difference between the two products is the number of struts from which they are braided. The PED stents consist of 48 struts, while P64 stents are made of 64 struts, including two thicker, so-called marker struts. If the results of this research are compared, in the nominally sized state P64 stents tend to have higher hydrodynamic resistances, PD and MSA values at a given DLR compared to PED stents. It can be seen in Fig. 2a, d, that from the two types of FDs, the P64 has around 40% larger pressure drop, if the FDs are nominally sized. It is also conspicuous in Fig. 2 that the P64 devices cover a wider HR range. Although these graphs show similar results to Gascou et al. [26], their measurement technique ignored the effect of the deployment length, which was shown previously to play an important role.

However, Fig. 2a, d shows that when oversizing the stents the pressure drop of P64 stents tends to decrease faster with DLR than PED stents. This difference can be explained through the theoretical relationship between the MSA and PD of a stent (Fig. 4). As seen from the continuous and dashed lines, as the MSA decreases, the theoretical value of the PD decreases faster in the case of P64 devices. The larger number of struts in the P64 devices cause the PD to decrease faster as the angle of the rhombi increases, which is proportional to the DLR. Therefore, elongating the device will make the HR decrease faster in the case of the P64 FDs.

These results imply that before treating an aneurysm, these differences should also be taken into account when choosing the appropriate FD device. The geometrical data and the HR of deployed PED FDs tend to vary less with radial compression or elongation or even radial sizing and P64 devices seem to be more sensitive to longitudinal compression/elongation. Yet P64 FDs can produce larger resistances between the aneurysm sac and the parent artery. The results are in concordance with the findings of Cancelliere et al. [27], who actually described the same phenomenon; however, the effect of the deployment length was not taken into consideration in their research, which seems to be a major determining factor.

### Limitations

Our study has some limitations. One of them is the number of measurements. Most of the deployment scenarios have at least three measurements, which is a too small number to employ statistical methods. The relationships between different geometrical and hydrodynamic parameters can be detected and interpreted through these results but more points could highlight the tendencies better; however, the extremely time-consuming nature of the measurements limits the number of experiments. Measuring other manufacturer’s products would also supplement the picture.

Another limitation compared to real-life usage is the constant diameters and straightness of the holder tubes. Blood vessel diameters decrease continuously, while the holder tubes used during this research are distinct and their number is limited. A 1mm oversizing in this diameter range is a relatively large step. Investigating the HR of deployed stents in curved holder tubes with different curvatures is planned in the future.

A further limitation is that our measurement technique refers to a resistance with perpendicular flow. In a real-life situation the flow direction varies in a wide range. Yet, one well-defined direction gives information on the tendencies, and effects of the parameters, even if the numerical values may vary with the flow angle.

Finally, it is worth noting that we do not state definite correlation between our measured parameters and the efficacy or safety of the flow diverter treatment. We quantified the effects of deployment techniques and sizing strategies to investigate the underlying mechanisms and aid neurointerventionalists in choosing the appropriate device and technique. It is up to the medical practitioners to translate these findings into therapeutic use.

## Conclusion

This study concentrated on providing objective quantitative results about the flow reducing capability of different flow diverter devices, which may have been known only qualitatively in clinical practice. We demonstrated that the deployment length is a major factor in determining the HR. By deploying the FD in an elongated fashion, its HR decreases. The PD and the MSA also change inversely with the deployment length. The radial sizing of the stents also plays an important role. Our results show that choosing an oversized device affects mainly the linear coefficient of the HR curve, while the quadratic coefficient is in the same range for the oversized and the nominally sized stents.

These results may represent a relevant quantitative piece of information for neurointerventionalists when choosing the flow diverter device for the treatment and the deployment length. The other significant result of this study is the coefficients of the HR curve, which are specific for the deployment technique and for the manufacturer. They can be used in patient-specific computational fluid dynamics simulations as coefficients of the porous layer for virtual stenting applications. The results presented in this article can be useful for both CFD simulations with the porous layer approach and physics-based clinical decisions.

## Supplementary Information

The Electronic Supplementary Material contains all measured data in two tables and the figures for the other deployment scenarios mentioned but not presented in the paper to provide further fundament to the described tendencies.
