# Supplementary material for: Impact of Design and Deployment Technique on the Hydrodynamic Resistance of Flow Diverters: An in Vitro Experimental Study
Source: Clin Neuroradiol. 2021 Oct 22;32(1):107–15. doi: 10.1007/s00062-021-01106-1 (PMC8894302; doi:10.1007/s00062-021-01106-1)
Supplement: Supplementary file 1 — The Electronic Supplementary Material contains all measured data in two tables and the figures for the other deployment scenarios mentioned but not presented in the paper to provide further fundament to the described tendencies. [file 62_2021_1106_MOESM1_ESM.pdf]

ONLINE RESOURCE MATERIAL 1

TITLE

Impact of design and deployment technique on the hydrodynamic resistance of flow diverters: an in vitro experimental study

Journal name

Clinical Neuroradiology

Corresponding author:

Dániel Gyürki

dgyurki@hds.bme.hu

OrcidID: 0000-0002-3124-808X

Department of Hydrodynamic Systems, Faculty of Mechanical Engineering, Budapest University of Technology and Economics, Budapest, Hungary

H-1111, Budapest, Műegyetem rkp. 3., D building, 3rd floor

Co-authors:

Benjamin Csippa

György Paál

István Szikora

Supplementary Table 1 Measured data for the nominally sized cases

	Manuf.	Size	$a\left[\frac{Pa\,s^2}{ml^2}\right]$	$b\left[\frac{Pa\,s}{ml}\right]$	DLR	MSA	$PD\left[\frac{pores}{mm^2}\right]$	$\alpha\,[°]$
FD5 in Tube5	PED	5x20	0.043	2.4	0.75	0.429	26	22
		5x20	0.050	1.5	0.78	0.344	18	28
		5x20	0.036	2.0	0.83	0.368	21	27
		5x20	0.028	2.0	0.85	0.432	22	22
	P64	5x24	0.213	4.4	0.58	0.481	34	27
		5x24	0.192	2.2	0.67	0.411	30	32
		5x24	0.035	2.2	0.83	0.385	27	37
FD4 in Tube4	PED	4x20	0.041	2.8	0.69	0.387	24	30
		4x20	0.055	3.1	0.71	0.357	21	32
		4x20	0.094	1.6	0.76	0.333	19	35
		4x20	0.072	1.3	1.12	0.283	16	46
	P64	4x24	0.126	5.0	0.64	0.449	32	31
		4x24	0.122	4.1	0.71	0.433	34	36
		4x24	0.065	2.7	0.92	0.369	28	47
FD3 in Tube3	PED	3x20	0.234	9.3	0.83	0.319	19	40
		3x20	0.017	4.5	0.87	0.309	17	43
		3x20	0.024	4.4	0.92	0.305	18	49
	P64	3x15	0.282	6.0	0.93	0.356	30	51
		3x15	0.058	3.5	0.97	0.357	29	56
		3x15	0.293	6.4	0.98	0.357	32	56

Supplementary Table 2 Measured data for the oversized cases

	Manuf.	Size	$a\left[\frac{Pa\,s^2}{ml^2}\right]$	$b\left[\frac{Pa\,s}{ml}\right]$	DLR	MSA	$PD\left[\frac{pores}{mm^2}\right]$	$\alpha\,[^\circ]$
FD5 in Tube4	PED	5x20	0.063	0.36	1.69	0.237	16	54
		5x20	0.104	0.29	1.83	0.204	12	75
		5x20	0.104	0.35	1.85	0.200	11	75
		5x20	0.106	0.64	1.90	0.200	11	77
		5x20	0.026	0.11	2.03	0.204	12	83
		5x20	0.018	0.26	2.03	0.199	10	83
	P64	5x24	0.014	0.16	1.19	0.270	20	73
		5x18	0.118	0.37	1.22	0.256	18	75
		5x24	0.084	0.14	1.29	0.261	21	75
		5x18	0.140	0.58	1.31	0.259	18	76
		5x18	0.104	1.01	1.35	0.258	18	75
FD4 in Tube3	PED	4x20	0.069	0.88	1.70	0.275	16	63
		4x20	0.061	0.75	1.83	0.239	13	68
		4x20	0.294	3.00	1.83	0.209	13	90
		4x20	0.025	2.00	1.90	0.205	13	89
	P64	4x18	0.308	3.51	1.44	0.263	18	90
		4x18	0.285	4.66	1.47	0.262	19	91
		4x18	0.091	1.55	1.51	0.259	18	91

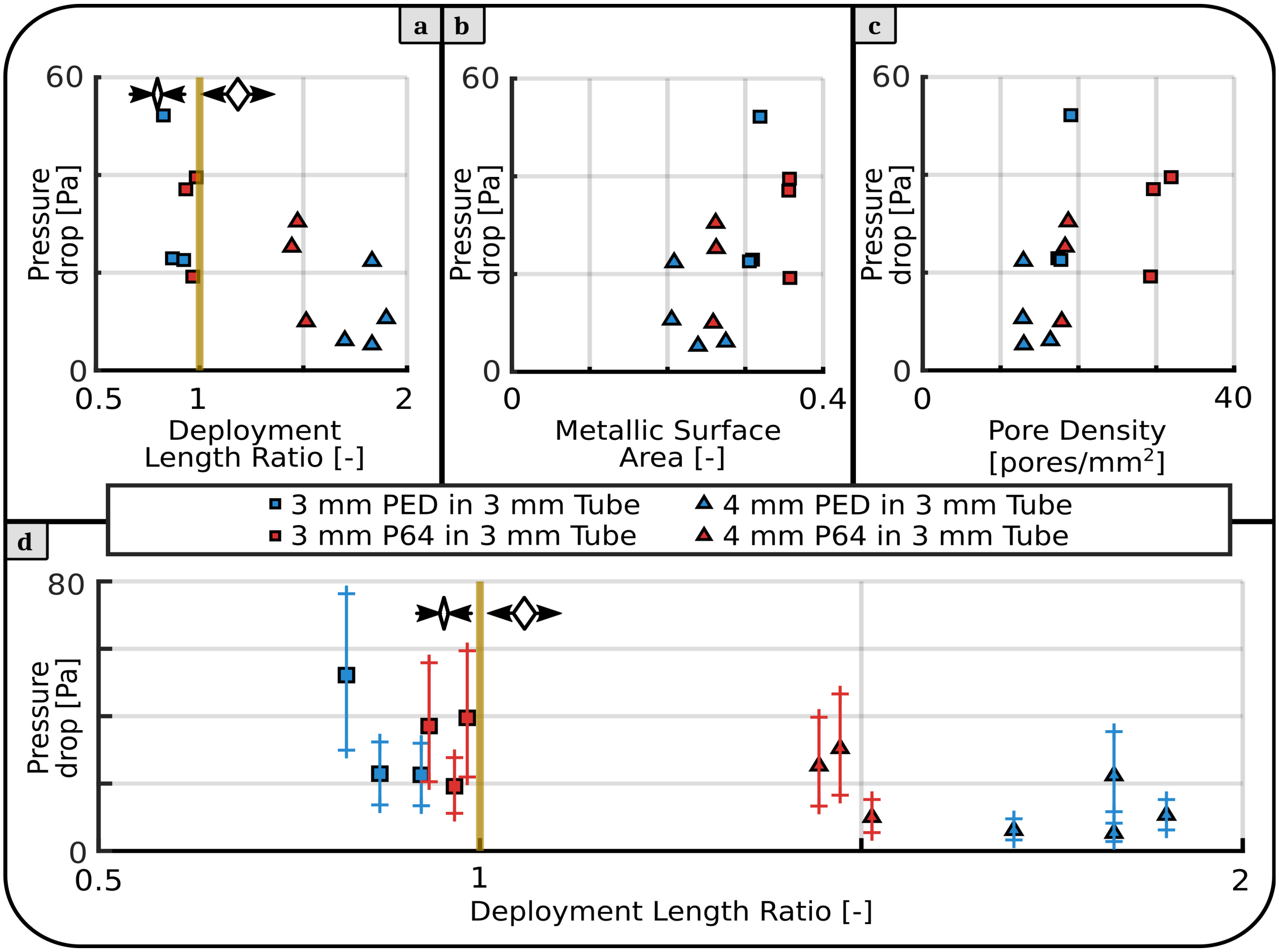

Supplementary Fig. 1 The effects of radial sizing in the case of FD4 and FD3 in Tube3 measurements. The vertical yellow lines represent the nominal deployment length, the small pictograms represent the longitudinally compressed and elongated deployment scenarios. Blue markers correspond to PED, while red markers correspond to P64 measurements. Squares are the nominally sized and triangles are the oversized cases

(a) Pressure drop calculated with five ml/s flow rate as the function of the deployment length ratio

(b) Pressure drop calculated with five ml/s flow rate as the function of the metallic surface area

(c) Pressure drop calculated with five ml/s flow rate as the function of the pore density

(d) Pressure drop calculated with five, three and seven ml/s flow rate (the mid-point and the two endpoints of the range, respectively, indicated by the symbol and the range around it) as the function of the deployment length ratio

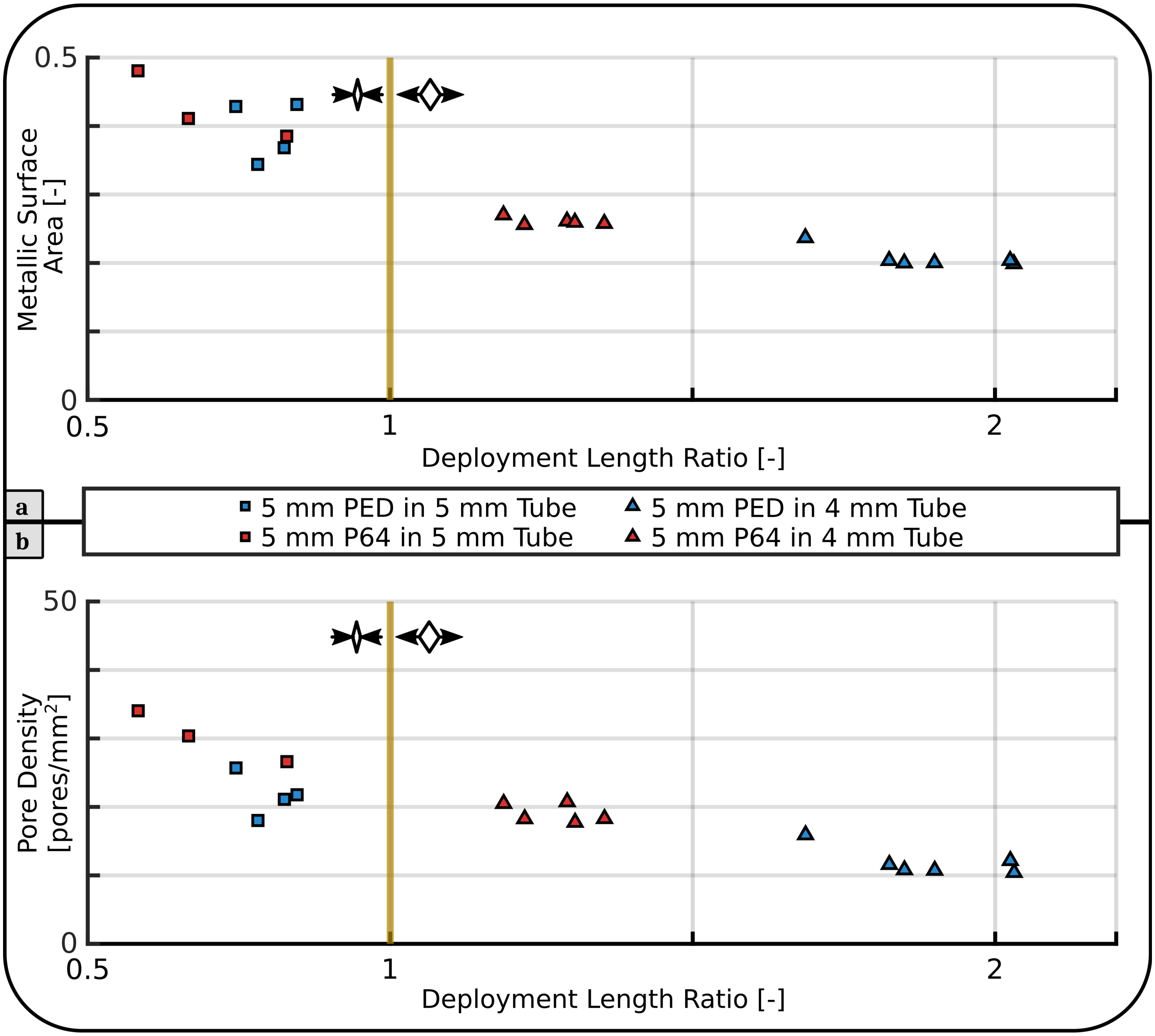

Supplementary Fig. 2 The effects of longitudinal compression or elongation in the cases of FD5 in Tube5 and Tube4 measurements. The vertical yellow lines represent the nominal deployment length, the small pictograms represent the longitudinally compressed and elongated deployment scenarios. Blue markers correspond to PED, while red markers correspond to P64 measurements. Squares are the nominally sized and triangles are the oversized cases

(a) Metallic surface area as the function of the deployment length ratio

(b) Pore density as the function of the deployment length ratio

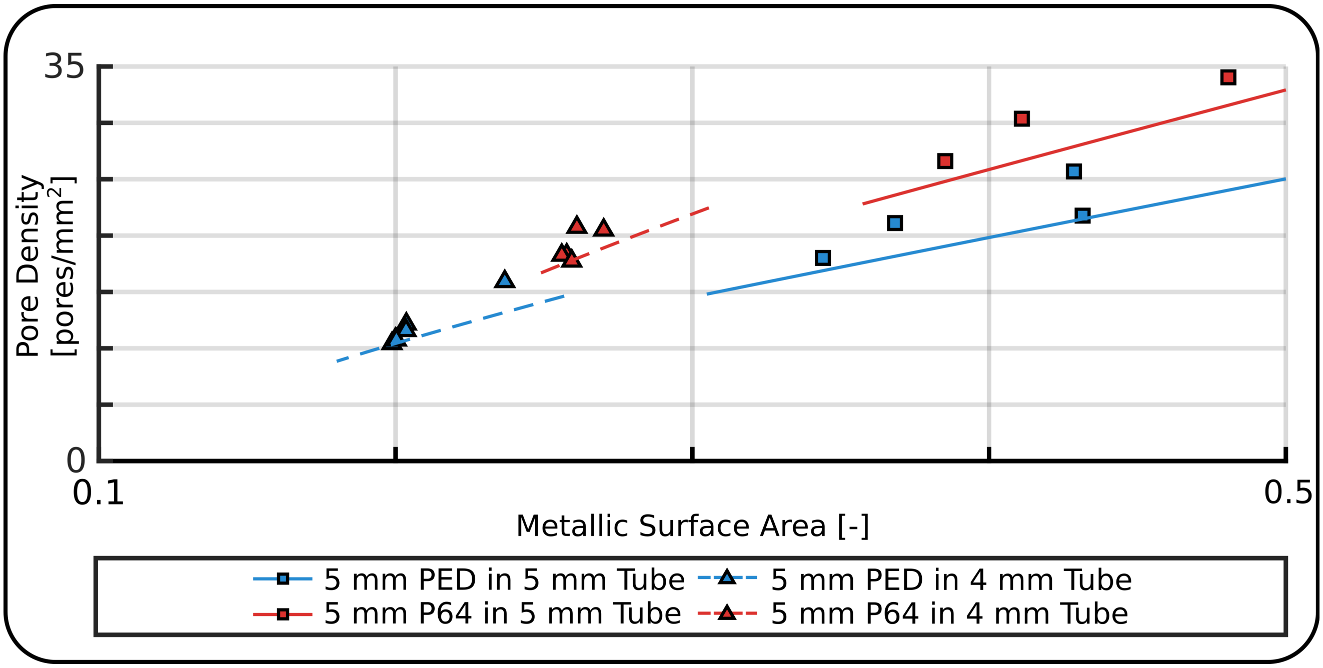

Supplementary Fig. 3 Pore density as the function of the metallic surface area in the cases of FD5 in Tube5 and Tube4 measurements. Blue markers correspond to PED, while red markers correspond to P64 measurements. Squares are the nominally sized and triangles are the oversized cases. The continuous and dashed lines represent the theoretical relationship calculated with devices diameter of five and four mm respectively
